# Advances of oncolytic vaccinia viruses armed with interleukin in tumor therapy

**DOI:** 10.3389/fonc.2025.1594621

**Published:** 2025-05-21

**Authors:** Mingyong Zha, Fei Huang, Songlin Li, Qi Wang, Yong Tang

**Affiliations:** ^1^ Department of Urology, Wuming Hospital of Guangxi Medical University, Nanning, Guangxi, China; ^2^ Department of Urology, The Second Affiliated Hospital of Guangxi Medical University, Nanning, Guangxi, China; ^3^ Department of Experimental Research, Guangxi Medical University Cancer Hospital, Nanning, Guangxi, China; ^4^ Key Laboratory of Early Prevention and Treatment for Regional High-incidence Tumors, Ministry of Education Key Laboratory, Guangxi Medical University, Nanning, Guangxi, China; ^5^ University Engineering Research Center of Oncolytic & Nanosystem Development, Nanning, Guangxi, China; ^6^ Department of Urology, Guangxi Medical University Cancer Hospital, Nanning, Guangxi, China

**Keywords:** gene therapy, interleukins, tumor microenvironment, oncolytic vaccinia virus, combined therapy

## Abstract

Oncolytic vaccinia viruses armed with interleukins represent a promising frontier in tumor therapy. Oncolytic vaccinia viruses express IL-2, IL-10, IL-12, IL-15, IL-21, IL-23, IL-24, and IL-36γ remodel the tumor microenvironment,enhance immune cell infiltration, suppress immunosuppressive elements and promot systemic antitumor immunity. Combinatorial strategies with chemotherapy, radiotherapy, metabolic modulators, immune checkpoint inhibitors, or natural compounds amplify therapeutic efficacy for tumors. In addition, we review the existing solutions to the problems of the immune clearance of virus, such as the use of inhibitors to prevent neutralizing antibodies from binding to the virus and the use of polymer encapsulation or mesenchymal stem cell loading. We also discussed Current directions include optimizing delivery systems, leveraging Artificial Intelligence for personalized designs of Oncolytic vaccinia virus inserted interleukins to guide the research in the future.

## Graphical Abstract

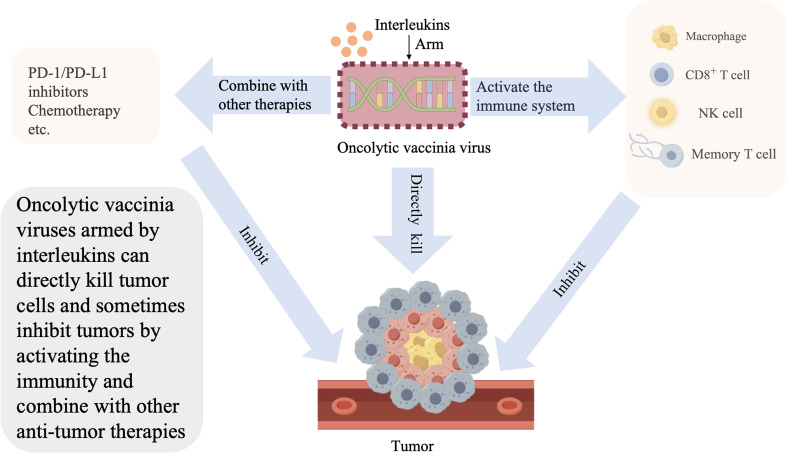

## 1 Introduction

Gene therapy is currently regarded as a promising strategy for the treatment of advanced malignant tumors. In this treatment modality, tumor cells can be killed directly by the therapeutic genes or cleared through the activation of immunity. Although wild oncolytic viruses (OVs) were initially utilized in cancer treatment in 1950, their use was discontinued because of low infection efficiency and poor anti-tumor effects (1). In 1991, Martuza et al. began to use engineered OVs with modified genomes (2), offering new promise for this type of therapy. The modified strategies of these OVs include the following: deleting unnecessary genes; weakening pathogenicity; enhancing the clearance of tumors; adding costimulatory molecules to the viral genome to improve antigen presentation and T cell activation, thereby enhancing the anti-tumor immune response; adding chemokines to the viral genome to improve the migration and infiltration of immune cells, as well as transform “cold” tumors into “hot” tumors; and adding cytokines to the viral genome to enhance the anti-tumor immune response (3). Therefore, inserting exogenous genes into the genome is an effective way to modify OVs.

OVs include vaccinia virus, herpes simplex virus, adenovirus, coxsackievirus, measles virus, and vesicular stomatitis virus. Gene insertion into RNA viruses (i.e., coxsackievirus, measles virus, and vesicular stomatitis virus) is difficult (4, 5). DNA viruses (i.e., herpes simplex, adenovirus, and vaccinia) can be modified by exogenous genes (6).Interleukins are widely present in the human body and profoundly affect tumor growth and metastasis. They have been found to remodel the tumor microenvironment (TME), promote the infiltration of immune cells, or directly kill tumor cells (7–9). Consequently, interleukins are used to arm OVs.

### 1.1 Limitations of herpesvirus/adenovirus modified by interleukins

Oncolytic adenovirus (OAd) is widely used with mature production technology and easy genome editing. Interleukins, along with other cytokines (chemokines, interferons, etc.), are used to modify OAd. However, the OAd genome is unable to accommodate multiple interleukins due to its small size (approximately 26–46 kb) (10). Due to the poor targeting of OAds, the carried interleukin is easily spread to the periphery. Consequently, the application of interleukin-modified OAds is limited. In addition, when OAd is attenuated, it reduces the immune response and virus replication (10–14).

Owing to its large genome, oncolytic herpes simplex virus (oHSV) has apparent advantages with regard to genetic modification. Several oHSVs have been applied to different types of cancer (15). Few studies have been conducted on modifying oHSV with interleukins, mainly focusing on interleukin-2 (IL-2),IL-12, IL-15, etc. (16–18). An oHSV expressing IL-2 (G47Δ-mIL2) enhances anti-tumor immune responses by releasing IL-2 locally. In a glioblastoma (GBM) mouse model, G47Δ-mIL2 significantly prolonged median survival without systemic IL-2 toxicity (18).Another important study by a Chinese team focused on VG161(an engineered oHSV that expresses IL-12, IL-15, IL-15Rα and a PD-1-PD-L1-blocking fusion protein) in the phase I clinical trial of refractory hepatocellular carcinoma showed that there was no dose-limiting toxicity in 44 treated patients with refractory hepatocellular carcinoma, and the main side effect was fever (86.4%). The objective response rate was 18.92% (7/37 cases had significant tumor reduction). The median progression-free survival was 2.9 months, and the overall survival was 12.4 months. The effect was even better in patients who had received prior immune checkpoint inhibitor therapy for more than 3 months, with median overall survival doubling (17.3 vs. 7.4 months). In addition, it was found that the necrosis and shrinkage of tumor lesions in the non-injected area were better than those in the injected area, suggesting that VG161 may exert anti-tumor effects by activating systemic immunity (19), In this study,VG161 was administered by intratumoral injection, but due to the widespread infection of herpes viruses in the population, it is still necessary to focus on the problem of immune clearance during systemic administration of oHSV.

### 1.2 The advantages of oncolytic vaccinia virus modified by interleukins

Oncolytic vaccinia virus (OVV) can accommodate large fragments of exogenous DNA for insertion without affecting its biological activity. Moreover, it offers genetic stability, rapid iteration with mature progeny produced in 6–8 h, a wide range of infectious forms, a variety of infected tumor cells, no need for a specific cell-surface receptor, great viability, and excellent clinical safety (20–26).OVVs can also directly lyse tumor cells, cause vascular damage, counteract the immune suppressive effects of the TME, and increase the infiltration of lymphocytes in the tumors. However, when used alone, unarmed OVVs have limited impact on tumors. In addition, they cannot effectively deal with tumor recurrence (26). Modification of OVVs involves the deletion of thymidine kinase to reduce its replication in normal cells and improve its targeting of tumor cells, deletion of NIL, VGF, Spi-1/2, A53R, A52R, E3L, F1L, K1L, etc., and insertion of exogenous genes (mainly cytokines) (26, 27), Interleukins are critical cytokines in arming OVVs (28).

The more complex effects of interleukins on immunity are gradually identified in studies. IL-2, IL-10, IL-33, and IL-21 can promote tumor growth and metastasis (29–34).However, the efficacy of IL-15, IL-8, and IL-17A/F, which have been utilized in clinical practice, is limited (35–38), Furthermore, IL-2 may also cause side effects, such as chills, high fever, capillary leakage, respiratory distress, liver and kidney damage, etc. (39).IL-2 demonstrated its therapeutic potential through persistent complete response in melanoma (40). Thus, the trend of research has shifted from interleukin monotherapy to the design of tumor-targeting fusion constructs aimed at improving efficacy and reducing systemic toxicities. Previous studies have shown that constructing a model of OVV expressing interleukins can significantly enhance the effects of anti-tumor immunity and confine interleukins to the TME, thus reducing the dose of interleukins and the unacceptable systemic toxicities. The combination of these two compensates for each other’s shortcomings. However, some problems, such as accelerating virus clearance, activating the signal transducer and activator of transcription 3 (STAT3) pathway, and recruiting regulatory T (Treg) cells, remain to be overcome.

In this article, we reviewed the mechanism underlying the effects of different interleukins, outlined the application of interleukin-armed OVVs, and analyzed the current problems of armed viruses. Moreover, we examined the possibility of combining interleukin-armed OVVs with other immunotherapies to promote the development of such models for clinical application (**Table 1**).

**Table 1:** Comparison of three oncolytic viruses.

Name of virus	Adenovirus	Vaccinia virus	Herpesvirus
Genome	dsDNA	dsDNA	dsDNA
Gene modification	E2F‐1,E1A,etc	TK,N1L,VGF,Spi-1/2 A53R,A52R,E3L,F1L,K1L,etc	UL56,ICP34.5,ICP6,etc
Gene insertion	Cytokine	Cytokine	Cytokine
Size	26~48kb	180~220kb	120~150kb
Capacity of inserted DNA	7.5kb	25∼40kb	30∼40kb
Advantages	High viral titers Editable genome	Excellent safety Capable Insertion of large genomes and multiple exogenous genes High viral titers Rapid replication	Capable Insertion of large genomes and multiple exogenous genes
Disadvantage	Poor targeting Difficult to insert large genomes and multiple exogenous genes	Easily cleared by immunity	Easily to cause infection Easily cleared by immunity
References	(10–14)	(20–28)	(15–19)

TK, thymidine kinase.

## 2 Interleukin-armed oncolytic vaccinia viruses

### 2.1 IL-2

The pleiotropic cytokine IL-2 is produced mainly by CD4^+^T cells, while a few are made by CD8^+^T cells, natural killer (NK) cells, and dendritic cells (DCs). It is a potent T cell mitogen and activator, which can extend the function of T cells and improve the immune clearance of tumors (41, 42). IL-2 binds with its receptor IL-2R, mainly acting on lymphocytes to activate the cytoplasmic adaptors Janus kinase 1 (JAK1) and JAK3, as well as the downstream signal pathways (e.g., STAT5A/B, STAT3/1), which further promote the activation and proliferation of lymphocytes. The differentiation and proliferation of DCs can be upgraded (43). However, IL-2 acts as a double-edged sword in immunity. It is proven to be a highly effective immunosuppressant. Treg cells are incredibly dependent on IL-2. It promotes the maturation of thymic Treg cells and peripheral Treg cells, which play an immunosuppressive role (44–49). Further investigation found that Treg cells express an IL-2 receptor trimer (CD25, CD122, CD132), indicating that Tregs tend to have a higher affinity for IL-2 (50, 51).In contrast, cytotoxic T cells and NK cells express IL-2 receptor dimers with only moderate affinity (52).In other words, low doses of IL-2 tend to induce Treg cell maturation to suppress immunity (53, 54), and only high doses can cause CD8^+^T cells and NK cells to mature and kill tumor cells (55–58).

Phase 3 clinical trial showed that the median survival time of patients with advanced melanoma who received high doses of intravenous IL-2 reached 25.8 months compared with 18.9 months recorded in the ipilimumab group. Treatment with high-dose IL-2 exerted a better effect. However, grade ≥3 adverse events occurred in all patients who received high-dose intravenous IL-2. High doses of IL-2 may be associated with high rates of side effects, including chills, high fever, capillary leakage, arrhythmias, respiratory distress, and renal insufficiency (59, 60). Therefore, it is desirable to develop an appropriate delivery method for maintaining IL-2 into the TME (61).

Liu et al. used OVV to load IL-12 and constructed a secreted form of vvDD-IL-2 and a membrane-bound form of vvDD-IL-2-RG; vvDD-IL-2 was markedly more toxic than vvDD-IL-2-RG. While the latter shows better efficacy, the survival of mice with colon and ovarian cancer was significantly prolonged, the levels of tumor necrosis factor-α (TNF-α) (a known toxic mediator of IL-2) were reduced, the tumor-infiltrating CD8^+^ T cell population was increased, and the CD8^+^/Treg cell ratio was increased significantly. The levels of interferon-γ (IFN-γ), granzyme B perforin, and T helper 1 cell-type (Th1 cell-type) chemokine C-X-C motif chemokine ligand 9 (CXCL9) were increased, whereas those of transforming growth factor-β (TGF-β) and angiogenesis markers (CD105 and vascular endothelial growth factor [VEGF]) were decreased. These results suggested that vvDD-IL-2-RG enhanced anti-tumor immunity and improved survival in mice (62).

IL-2 combined with cisplatin is effective in treating primary and secondary malignant pleural effusion (63). Ekeke et al. constructed an OVV expressing yellow fluorescent protein (VV-YFP) and another expressing IL-2 (VV-IL-2). VV-IL-2 significantly reduced the tumor burden and improved the survival rate of malignant pleural disease mice, had no significant systemic toxicities compared with VV-YFP, and led to an increase in tumor-infiltrating lymphocytes, particularly CD8^+^T cells (64).

### 2.2 IL-10

IL-10,secreted by macrophages, T cell subsets and NK cells (65),; its role in tumors is controversial. Evidence indicates that IL-10 can help tumor cells escape immune surveillance (31, 32). High levels of IL-10 are found in the tumors and serum of numerous patients with malignancies (66–70). IL-10 promotes tumor growth and metastasis in many ways, including downregulation of antigen-presenting cells and major histocompatibility complex (MHC) expression in tumors to reduce antigen presentation (32, 71),promote naive CD4^+^ T cells to Th2 cells (72), and encourage the loss of E-calmodulin and increase of N-calmodulin (73), thus promoting tumor growth. IL-10 binds to the receptor IL-10R expressed by tumor cells, activating JAK1 and tyrosine kinase 2 (TYK2); this is followed by activation of STAT3 (74), which is involved in the growth and metastasis of tumors (75–78).

IL-10 promotes melanoma growth by stimulating angiogenesis and immunosuppression in mice (79). It also encourages the proliferation of glioma cells *in vitro* (80). Accordingly, IL-10 alone may not be effective against tumors. It has been reported that IL-10 enhances the inhibitory effect of OVV on tumor cells in mice (81). However, the experiment only applied IL-10 after the injection of OVV and did not use OVV as a vector. Inserting IL-10 into the OVV genome may be more promising.

Chard et al. constructed two models of OVV, namely VVLΔTK-IL-10 and VVLΔTK. In a mouse model of pancreatic cancer, 87.5% of mice treated with VVLΔTK-IL-10 produced complete tumor clearance versus 40% of mice treated with VVLΔTK. In the model of advanced pancreatic cancer *in situ*, the survival time of mice treated with VVLΔTK-IL-10 was significantly improved to 138.5 days, compared with 69.7 days after VVLΔTK treatment (P<0.01). Moreover, IL-10 reduced viral clearance and tumor recurrence, confirming the potential of VVLΔTK-IL-10. Although VVLΔTK-IL-10 treatment reduced the antiviral CD8^+^ T cell population, an increase in anti-tumor CD8^+^ T cells was observed at some time. This finding suggested that IL-10 reduced viral clearance, but did not suppress anti-tumor immunity. Research has shown that macrophages present viral antigen and IL-10 downregulates MHC-II of macrophages, inhibits the antigen-presenting ability of macrophages, decreases viral clearance, and increases tumor cell infection and tumor cell antigen release. Therefore, IL-10-armed OVV shows promise as a novel treatment for pancreatic cancer by enhancing tumor inhibition through the regulation of innate and adaptive immune responses (82, 83).

### 2.3 IL-12

IL-12 is a proinflammatory cytokine secreted by activated antigen-presenting cells (84, 85). IIt is a dimeric factor composed of IL-12B (P40) and IL-12A (P35) (84), which bind to the receptors of IL12 (IL-12Rβ1 and IL-12Rβ2) and activate TYK2 and JAK2, eventually leading to the phosphorylation of STAT4 and the production of IFN-γ. IL-12Rβ1 and IL-12Rβ2 are primarily expressed in NK cells and CD8^+^T cells, and the activation increases their proliferation (86, 87). In addition, IL-12 can induce Th1 differentiation, reducing the expansion of immunosuppressive cells such as tumor-associated macrophages and myeloid-derived suppressor cells (MDSCs) (88–90).Furthermore, IL-12 promotes the production of more IFN-γ by CD8^+^ T cells, exerting its inhibitory effect on tumors. It can inhibit tumor angiogenesis while upregulating MHC-I and MHC-II expression in tumor cells, thereby improving the effectiveness of their recognition and elimination (85, 91, 92).

However, even though IL-12 showed potent anti-tumor effects in pre-clinical trials, its impact in clinical trials was negligible at tolerable doses (93–95). In addition, systemic administration of IL-12 is extremely toxic (96, 97). Hence, the application of new delivery modes should be a top priority for research.

Ge et al. constructed membrane-bound OVV vvDD-IL-12-FG and secreted vvDD-IL-12. Mice treated with vvDD-IL-12 had significantly higher levels of IL-12 in tumors than those treated with vvDD-IL-12, and vvDD-IL-12 induced pulmonary and renal edema in mice. In contrast, vvDD-IL-12-FG did not exert these toxic effects and effectively immobilized IL-12 in the TME. Moreover, vvDD-IL-12-FG had the same anti-tumor effect. CD4^+^ and CD8^+^ T cells and IFN-γ were increased in tumors of vvDD-IL-12-FG-treated mice. In addition, vvDD caused an increase in MSDCs while vvDD-IL-12-FG inhibited MSDCs and Treg cells, leading to a significant decrease in the expression of TGF-β, cyclooxygenase-2 (COX-2), and angiogenesis markers (VEGF and CD105). This suggests that vvDD-IL-12-FG can deliver IL-12 to the tumor, allowing IL-12 to exert its efficacy without being limited by safety concerns (98).

AZD4820, an OVV that expresses IL-12, showed a complete response in 60% of mice treated with AZD4820 in a mouse model of colon cancer, while no mice showed a complete response (CR),after treatment with the original virus strain. AZD4820 treatment, in combination with PD-L1 blocking antibodies, enhanced tumor-specific T-cell immunity relative to monotherapy. These findings suggest that vaccinia virus delivery of IL-12, combined with immune checkpoint block, induces anti-tumor immunity in tumors that do not respond well to immune checkpoint inhibitors(ICIs) (99).

### 2.4 IL-15

As a member of the IL-2 family, IL-15 plays a similar role to that of IL-2. Although IL-15 can regulate innate and adaptive immunity, it causes unacceptable toxicity when administered systemically (100). The specific receptor of IL-15 (IL-15Rα) is secreted primarily by NK, T, and B cells (101).Binding of IL-15R to IL-15Rα produces the IL-15R/IL-15Rα complex, which binds to the IL-2Rβγ (IL-15Rβγ) expressed by effector cells (NK, T, and B cells), and further activates them to kill tumor cells (102). IL-15 appears less harmful to the TME than IL-2, as it fails to activate Tregs in a similar manner to IL-2, and occasionally inhibits Tregs (103).IL-15 may be more efficient than IL-2 in tumor suppression. However, IL-15 causes systemic toxicity and is ineffective in head and neck cancer and lung cancer, promoting tumor progression (104, 105). This observation may be related to the short half-life of IL-15 *in vivo*, elevation of the nflammatory cytokines IL-6 and IFN-γ, etc. (106). Application of IL-15 caused peripheral NK cell activation, which in turn induced IFN-γ expression, leading to systemic toxicity and tumor progression (100). OVVs can immobilize IL-15 in the TME. The short half-life and low biological activity of IL-15 *in vivo* can be addressed by applying the fully soluble IL-15Rα as an IL-15 agonist (107). Moreover, the effect of the IL-15Rα/IL-15 complex is relatively long-lasting.

Kowalsky et al. inserted the mouse IL-15-IL-15Ra fusion gene into OVV and named it vvDD-IL15-Rα. vvDD-IL15-Rα significantly inhibited colon and ovarian cancer in mice, which progressed rapidly in the vvDD group. Of the mice treated with vvDD-IL15-Rα, 80% were cured with no tumor recurrence; IFN-γ and tumor-specific CD8^+^T levels were increased. When rechallenged by tumor cells, the mice completely resisted recurrence. These findings indicate that vvDD-IL15-Rα can effectively stimulate anti-tumor adaptive immunity. However, vvDD accumulates more effectively in the tumor than vvDD-IL15-Rα (108). Considering that the researchers did not detect the total number of CD8^+^T cells and the number of virus-specific CD8^+^T cells, it is impossible to determine whether the superagonist proteins clear the virus by virus-specific CD8^+^T cells. This should be investigated in studies in the future. The antiviral effect of IL-15 may be dependent on the presence of IFN during OVV infection (109), and IFN is strongly upregulated during VVDD-IL15-Rα application (108). This may explain the mechanism through which superagonist proteins clear vvDD-IL15-Rα.

Shakiba et al. constructed armed OVVs based on IL-15 and its receptor, respectively, and inserted red fluorescence protein (RFP); the constructs were named LIVP-IL15-RFP and LIVP-IL15Ra-RFP, respectively. By simultaneous use of these two armed viruses, IL-15 can bind to IL-15R *in vivo*, form an IL-15/IL-15R complex to maintain the effect of IL-15 on the TME, and limit its toxicity. In mice with colon cancer and breast cancer, the combination of LIVP-IL15-RFP and LIVP-IL15Ra-RFP led to the lowest rate of tumor progression and the highest survival rate compared with any monotherapy. The LIVP-IL15-RFP group had an increase in C-C motif chemokine ligand 2 (CCL2), VEGF, and IL-4 inflammatory cytokines, suggesting an IL-15-based side effect. In contrast, these levels were within normal ranges in the combination group. The significant increase in IFN-α, IFN-γ,granulocyte-macrophage colony stimulating factor (GM-CSF), and TNF-α suggested a more effective anti-tumor effect in the combination group (110).

### 2.5 IL-21

As a member of the IL-2 family, IL-21 is a pleiotropic cytokine secreted by activated CD4^+^ T cells and NK cells that regulates lymphoid and myeloid cells (111–113). Its receptor IL-21R is expressed by various immune cells, such as T cells, B cells, NK cells, DCs, and intestinal epithelial cells (non-immune cells) (114). Therefore, IL-21 may have a broad and powerful effect on immunity. IL-21R is a trimer consisting of γc, a unique α-chain (CD25), and a β-chain (CD122). Binding of IL-21 to the IL-21Rα/γc complex activates the JAK1 and JAK3 pathways (115),. This is followed by phosphorylation of STAT1/3 downstream, and a slight activation of STAT5 (116). Sustained STAT3 activation leads to the proliferation of many tumor cells (75–78).The widespread expression of IL-21R in immune enables IL-21 to activate macrophage, DCs, NK cells, B cells, NK T cells, CD4^+^T cells, CD8^+^ T cells, Treg cells, Th17 cells, follicular helper T cells, etc. (78, 113, 117–122). From this perspective, IL-21 appears to have a complex role in the TME. Although current data show that IL-21 has acceptable toxicity (123, 124), and its efficacy against tumors is fine (112), its ability to recruit cytotoxic T cells alone remains insufficient. Thus, a combination with OVVs may potentiate the effectiveness of IL-21.

Kowalsky et al. constructed an IL-21-armed OVV rTTVΔTK-mIL21, which was more effective in suppressing injected and distant tumors than rTTVΔTK and phosphate-buffered saline (PBS). Treatment with rTTVΔTK-mIL21 prolonged the survival of melanoma and glioma mice and increased NK cells, CD3^+^ T cells, CD4^+^ T cells, and CD8^+^ T cells. In addition, the immune cells of distant tumors were increased, while viral infection was note detected. Tregs were inhibited to an extent which was equal to that noted in the PBS group. This suggests that rTTVΔTK-mIL21 induces more robust systemic immunity with less immune resistance (125).

Another study showed that VVLΔTKΔN1L-mIL-21, an OVV expressing IL-21, exhibited more potent anti-tumor effects than VVLΔTKΔN1L-RFP in a C57 mouse colon cancer model. The rate of complete tumor regression (CR) was 85.7% and 42.9% in the VVLΔTKΔN1L-mIL-21 group and VVLΔTKΔN1L-RFP group, respectively. Compared with the VVLΔTKΔN1L-RFP group, the VVLΔTKΔN1L-mIL-21 group had a slight increase in CD4^+^ and CD8^+^T cells and a significant increase in central memory T cells. These results suggested that VVLΔTKΔN1L-mIL-21 had a more potent inhibitory effect on tumor recurrence. Notably, VVLΔTKΔN1L-mIL-21 did not exhibit a more potent anti-tumor effect than VVLΔTKΔN1L-RFP, and replication was significantly reduced in a BALB/c mouse model of colon cancer. Thus, we can speculate the following: 1) VVLΔTKΔN1L has a poor therapeutic effect and affinity for colon cancer; 2) C57BL/6 mice and BALB/c mice have different genetic backgrounds and immune responses to the virus; 3) IL-21 may mediate antiviral immunity and lead to virus clearance; and 4) tumor heterogeneity is an essential factor affecting the efficacy of treatment. Therefore, it is crucial to develop personalized tumor treatment (126).

IL-21-armed OVV VΔTK-STCΔN1L-IL-21 was injected into subcutaneous, orthotopic and disseminated mouse models of pancreatic cancer, and phosphoinositide 3-kinase δ (PI3Kδ) inhibitor CAL-101 was used to inhibit the virus uptake by macrophages. Compared with VΔTK-STCΔN1L, CAL-101, and PBS, treatment with VΔTK-STCΔN1L-IL-21 did not reduce CD4^+^ cells. Nevertheless, it significantly increased CD8^+^T cells of circulation and tumors, as well as reduced CD8^+^T cells in the spleen and mobilized them to the tumors. The effector CD8^+^ T cells, central memory CD8^+^ T cells, and NK cells were increased in the circulation, spleen, and tumors. These observations suggested that VΔTK-STCΔN1L-IL-21 has the most apparent inhibitory effect on tumor recurrence and can activate innate and adaptive immunity (127).

### 2.6 IL-23

IL-23 belongs to the IL-12 cytokine family, which consists of IL-12B (P40) and IL-23P19 or IL-23A subunits. The IL-23 receptor is composed of IL-12Rβ1 and IL-23R. IL-12Rβ1 binds to TYK2 and induces STAT4 phosphorylation, which is critical for increased IFN-γ production and subsequent Th1 cell differentiation (128). IL-23R interacts with JAK2 to induce STAT3 phosphorylation and promote Th17 cell proliferation (129).

The role of IL-23 in the treatment of cancer appears to be controversial. It is generally thought that endogenous IL-23 promotes tumors, while exogenous IL-23 inhibits tumors (130). Endogenous IL-23 is secreted by myeloid cells, and mouse tumor cells do not secrete IL-23 (131)However, human tumor cells secrete a small amount of IL-23 (132). Considering that quantitative polymerase chain reaction and western blotting assays are mainly used, whether human tumors secrete bioactive IL-23 remains to be proven. Chen et al. constructed an OVV expressing IL-23, termed vvDD-IL-23, which has shown a better anti-tumor effect than vvDD in several mouse tumor models. In mouse models of colon cancer, vvDD-IL-23 induced IL-10 expression, inhibited antiviral immunity, upregulated the expression of Th1 chemokine, IFN-γ, TNF-α, perforin, IL-2, and GZMB, and regulated the TME compared with vvDD. The expression of immune checkpoint cytotoxic T-lymphocyte associated protein 4 (CTLA-4), PD-1, and PD-L1 continued to increase after vvDD-IL-23 treatment. This evidence provides a theoretical basis for the next application of armed OVVs in combination with ICIs. In advanced tumors, mice treated with vvDD-IL-23 developed long-lasting systemic anti-tumor immunity, significantly increasing CD4^+^ and CD8^+^ T cells in tumors, whereas Treg cells were unaffected (133).

### 2.7 IL-24

IL-24 is a member of the IL-10 cytokine family (134), produced by IL-4-inducible monocytes and Th2 lymphocytes. Previous studies have primarily focused on the killing effect of IL-24 on tumor cells. However, recent studies have shown that IL-24 also affects immunity. IL-24 has two receptors, namely IL-20R1/IL-20R2 and IL-22R1/IL-20R2 (135). Binding of IL-24 to these two receptors activates the JAK/STAT pathway, primarily STAT3. However, activation of the JAK/STAT pathway is not involved in the apoptotic signaling of tumors by IL-24. IL-24 focuses more on the secretion of its protein, which does not damage the normal cell. Its accumulation leads to an unfolding protein response and endoplasmic reticulum stress in tumor cells, ultimately inducing tumor cell apoptosis (136, 137). Thus, IL-24 has no significant side effects and does not cause a cytokine storm (138). IL-24 is thought to induce the expression of IFN-γ, TNF-α, and IL-6 (139)by lymphocytes, promote Th1-like cytokine secretion and the immunoreactivity of DCs, activate cytotoxic CD8^+^ T cells (140, 141), induce the proliferation of memory T cells, and reduce the number of Treg cells (140, 142).

Deng et al. constructed the OVV model VG9-IL-24. Compared with VG9-EGFP (the original strain of VG9 with insertion of the enhanced green fluorescent gene), VG9-IL-24 more significantly inhibited tumors and prolonged the survival of mice (143). Nonetheless, the effects of IL-24 on the TME were not thoroughly investigated in that study.

In a bilateral mouse model of colorectal tumor, the investigators focused on the effects of VG9-IL-24 on the TME, injecting VG9-IL-24 into the tumor on one side only. The results showed regression in both sides. IL-24 activated anti-tumor immunity and eliminated primary and distant tumors. Moreover, VG9-IL-24 can promote the secretion of IFN-γ and IL-6 at high levels, and TNF-α and IL-4 at low levels. These levels were significantly higher than those detected in the PBS and VGg-EGFP groups, suggesting that VG9-IL-24 induced specific anti-tumor immunity. In addition, IL-24 activates STAT3, which often predicts tumor progression, and VG9-IL-24 can counteract the tendency of STAT3 phosphorylation (144). However, the effects of VGg-IL-24 on various immune cells have not been clarified, and most of them are limited to the direction in which IL-24 can induce tumor cell apoptosis. The more far-reaching effects of IL-24-arming OVV on the immune system are worthy of further exploration.

### 2.8 IL-36γ

IL-36γ is a member of the IL-1 gene family. It binds to its receptors IL-36R (IL-1Rrp2) and IL-1RAcP to activate DCs, T cells, and NK cells (145, 146). IL-36γ alters the TME and promotes type 1 lymphocyte-mediated anti-tumor immunity (147). It has also been shown that IL-36γ induces colony formation, migration, and invasion of gastric cancer cell lines. In addition, the expression of IL-36γ was higher in primary gastric tumors compared with normal tissues (148).

Using IL-36γ alone in the treatment of tumors is controversial. The negative effects of IL-36γ on tumor immunity may be avoided by using it as an enhancer of OVVs. By inserting IL-36γ into three OVVs, Yang et al. constructed three models, namely vvTK-IL-36γ, vvDD-IL-36γ, and vvTD-IL-36γ. The results showed that vvTK-IL-36γ has a stronger anti-tumor effect than vvTK in a mouse model of colon cancer. Compared with vvTD, VVTD-IL-36γ significantly prolonged the survival of mice in models of pancreatic cancer and colon cancer. In the mouse model of colon cancer, the greatest numbers of CD8^+^ and CD4^+^ T cells were observed in the vvDD-IL-36γ group. Moreover, IL-36γ promoted the differentiation of naive CD8^+^ T cells into memory and effector T cells, and increased NK cells and DCs. Nevertheless, it decreased myeloid-derived suppressor cells and M2-like tumor-associated macrophages and increased tumor antigen-specific T cells (149). IL-36γ could enhance the activity of a variety of OVVs, and mice cured of colon cancer could cope with the dual challenge of colon cancer and lung cancer cells. These findings suggested that IL-36γ-armed virus could reverse the TME and activate systemic immunity.

## 3 Progress of interleukins together with other cytokines inserted into OVVs in tumor treatment

The hIL-7-VV (an OVV expressing human IL-7) and mIL-12-VV (an OVV expressing mouse IL-12) were constructed by Nakao et al. These OVVs did not show satisfactory anti-tumor effects when used alone. However, in the combination group, four of seven tumor-bearing mice achieved CR, and a greater number of CD8^+^ T cells, CD4^+^ T cells, NK T cells, and NK cells infiltrated the tumor. To reduce the dose of the virus, the researchers simultaneously inserted hIL-17 and mIL-12 into OVV to construct hIL-17/mIL-12-VV. This approach achieved excellent CR in various mouse tumor models. In a bilateral tumor model, injection of hIL-17/mIL-12-VV on one side resulted in tumor regression and immune cell infiltration on the other side with no virus detected. Based on these results, hIL-17/mIL-12-VV can inhibit metastatic tumors by activating systemic immunity, which has a positive significance for treating advanced tumors (150).

**Figure 1:**
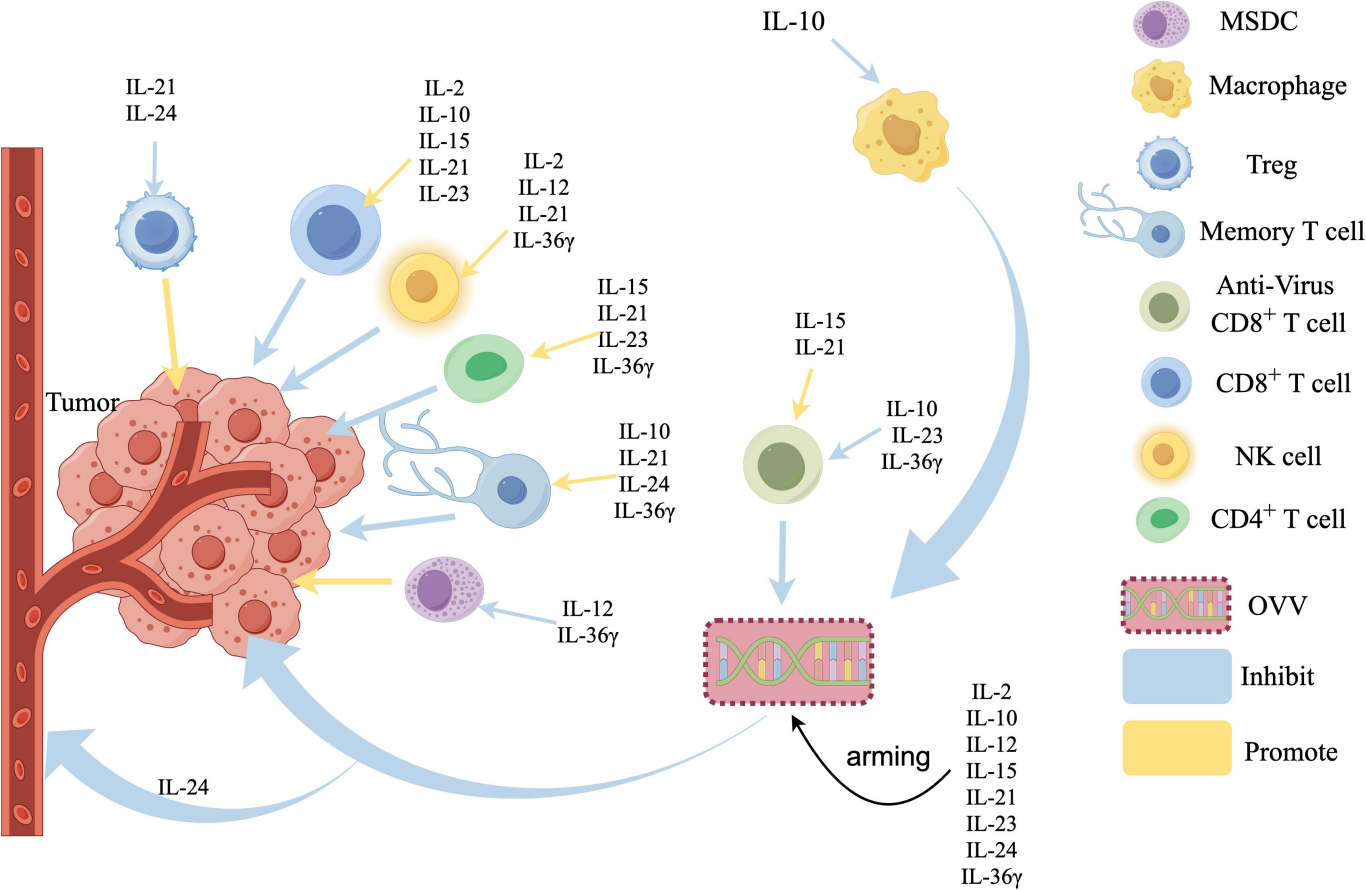
Effects of interleukin-armed OVVs on tumor microenvironment. IL-15, IL-21, IL-23, IL-36γ Promote the infiltration of CD4^+^ T cells in the tumors, IL-2, IL-12, IL-21, IL-36γ activate Natural Killer (NK) cells, IL-2, IL-10, IL-15, IL-21, IL-23 promote CD8^+^ T cells infiltration, IL-10, IL-23, IL-36γ inhibited anti-virus CD8^+^T cells, IL-15, IL-21 promote anti-virus CD8^+^T cells, IL-10, IL-21, IL-24, IL-36γ promote the production of Memory T cells, IL-21 and IL-24 inhibite regulatory cells (Tregs). IL-2 and IL-36γ inhibited Myeloid-derived suppressor cells (MDSCs), IL-10 inhibits Macrophages,IL-24 can directly inhibit the blood vessels.

In another study, an OVV based on the VG9 strain that co-expressed GM-CSF and IL-24 was constructed, namely VG9-GMCSF-IL24. Compared with VG9 and PBS, VG9-GM-CSF-IL24 showed superior anti-tumor effect in breast cancer, melanoma, and colorectal cancer, and the tumors secreted the highest levels of IFN-γ, TNF-α, IL-4, and IL-6 (151).Thus far, two genes inserted in OVV appear to offer a significant advantage and better tumor suppression effect than a single one. However, previous studies found that VG9-IL-24 could also induce the secretion of IFN-γ, TNF-α, IL-4, and IL-6 (144). Hence, it is important to understand the role that GM-CSF plays in the immune system, the mechanism through which it exert its anti-tumor effect, and whether it impacts the anti-tumor effect of IL-24.In addition, delivery of IL-7 or IL-12 alone is ineffective. This appears to be different from the effect of AZD4820, which also expresses IL-12. AZD4820 resulted in a CR of 60%, while mIL-12-VV led to a CR of only 14%.Be used in two different tumor models certainly should be considered in this situation, but hIL-7-VV only achieved a CR of 0. Furthermore, the combination of IL-7 and IL-12 resulted in a CR of 57%, and the CR linked to hIL-17/mIL-12-VV was even better (150, 152), the synergistic effect of the two genes is surprising, considering that IL-7 does noy play a positive role in the TME (153).The mechanism by which IL-7 and IL-12 interact to reverse their roles in the TME warrants further investigation (**Table 2**, **Figure 1**).

**Table 2:** Research on Interleukins armed oncolytic vaccinia viruses.

Interleukin	Original vaccinia viruses	Armed virus models	Cancers	Advantages	Disadvantages	References
IL-2	vSC20	vvDD-IL-2 vvDD-IL-2-RG VV-IL-2	Colon cancer Ovarian cancer Malignant pleural effusion	Lower side effects Better targetability Stronger anti-tumor effects More CTLs calling	Treg cells calling Activating STAT3	(62, 64)
IL-10	LIVP	VVLΔTK-IL-10	Pancreatic cancer	Better targetability Stronger anti-tumour effects Prevention of tumor recurrence More CTLs calling viral clearance delayed	Activating STAT3	(82, 83)
IL-12	vSC20	vvDD-IL-12-FG vvDD-IL-12	Colon cancer	Lower side effects Better targetability Stronger anti-tumour effects More CTLs calling Tregs, TAMs and MDSCs reduced		(98, 99)
IL-15	vvDD LIVP	vvDD-IL15-Rα LIVP-IL15-RFP IVP-IL15Ra-RFP	Colon cancer Ovarian cancer breast cancer	Lower side effects Better targetability Stronger anti-tumour effects More CTLs calling	viral clearance	(108, 110)
IL-21	TTV752-1 ListerVVΔTK VΔTK-STCΔN1L	rVVLΔTKΔN1L-mIL-21 VVLΔTKΔN1L-mIL-21 VΔTK-STCΔN1L-IL-21	Melanoma Glioma Colon cancer Pancreatic cancer	Better targetability Stronger anti-tumour effects More CTLs calling Prevention of tumor recurrence Tregs reduced	Activating STAT3 viral clearance	(125, 126)
IL-23	VSC20	vvDD-IL-23	Colon cancer Breast cancer Melanoma Lung cancer Ovarian cancer	Better targetability Stronger anti-tumour effects More CTLs calling viral clearance delayed	Activating STAT3	(133)
IL-24	VG9	VG9-IL-24 VV-IL24	Colorectal Cancer Hepatocellular carcinoma Lung cancer Breast cancer	Better targetability Stronger anti-tumour effect Prevention of tumor recurrence Tregs reduced	Activating STAT3 viral clearance	(143.144)
IL-36γ	vJS6 vSC20 vSPT	vvTK-IL-36γ vvDD-IL-36γ vvTD-IL-36γ	Colon cancer Pancreatic cancer	Better targetability Stronger anti-tumour effects More CTLs calling Prevention of tumor recurrence TAMs and MDSCs reduced		(149)

IL, interleukin; CTLs, cytotoxic T cells; Tregs, regulatory cells; TAMs, tumor-associated macrophages; MDSCs, myeloid-derived suppressor cells; STAT3, activator of transcription 3.

## 4 Progress of interleukin-armed oncolytic vaccinia virus combined with other therapies in tumor treatment

### 4.1 Progress of interleukin-armed oncolytic vaccinia virus combined with chemotherapy in tumor treatment

Thrombocytopenia is one of the most common side effects of the chemotherapeutic drug mitomycin C, and IL-6 is thought to promote platelet production (154); Thus, IL-6 can alleviate the side effects of mitomycin C and enhance its efficacy. Investigators constructed a hyper-IL-6 fusion protein to enhance the activity and effect of IL-6. The hyper-IL-6 encoded vaccinia virus GLV-1h90 was constructed based on the OVV strain GLV-1h80. GLV-1h90 showed similar anti-tumor effects to those of GLV-1h80 in a mouse model of prostate cancer, indicating that hyper-IL-6 may not have anti-tumor activity. However, the GLV-1H90-vaccinated mice were more active and gained weight, meaning they were healthier. Although it was more effective than any single agent, GLV-1h90 did not exhibit superior anti-tumor effects than GLV-1h80 in combination with mitomycin C. The combination of GLV-1h90 and mitomycin C alleviated the thrombocytopenia and accelerated recovery. These results suggested that GLV-1h90 potentiated mitomycin C and attenuated its side effects (155).

### 4.2 Progress of oncolytic vaccinia virus expressing interleukin combined with immune checkpoint inhibitors in tumor therapy

Numerous interleukins amplify immune checkpoint blockade. Moreover, OVs are thought to effectively improve the TME, attract immune cells, and upregulate the expression of programmed cell death-ligand 1 (PD-L1) in tumor cells, thereby amplifying the blockade of the programmed cell death-1/PD-L1 (PD-1/PD-L1) monoclonal antibody. Therefore, interleukins, OVs, and immune checkpoint blockade can be combined to maximize anti-tumor effects (9, 108, 156).

Treatment with vvDD-IL-2-RG has a poor therapeutic effect in mice with high tumor burden, but increases the expression of PD-1, PD-L1, and CTLA-4 in tumors. Hence, vvDD-IL-2-RG can be combined with anti-PD-1 and anti-CTLA4 to improve the prognosis of mice with advanced tumors. In the advanced colon cancer model, a combination of vvDD-IL-2-RG and anti-PD-1 cured most of the mice, activated systemic immunity, and suppressed distant tumors. However, the vvDD-IL-2-RG combined with anti-CTLA4 was not effective enough. We hypothesized that because the action of anti-CTLA4 is different from anti-PD-1, anti-CTLA4 mainly acts on CD4^+^ T cells at the initiation stage of immune response (157). In contrast, anti-PD-1/PD-L1 mainly acts on exhausted T cells within the tumor (158). Thus, vvDD-IL-2-RG recruited T cells to re-infiltrate, possibly amplifying the effect of anti-PD-1 (62).

In a mouse model of pancreatic cancer, the combination of VL-21 and PD-1 blockade significantly inhibited tumor growth and improved the overall survival rate compared with VL-21 alone. Greater numbers of CD8^+^T cells were also accumulated in the tumors (127).In glioma, VVLΔTK-STCΔN1L-mIL-21 can remarkably increase the expression of PD-L1 in tumor cells and amplify the therapeutic effect of anti-PD-1. In tumor-bearing mice (159).

In another study, vvDD-IL15-Rα combined with anti-PD-1 completely cured all the mice bearing colon tumors and prolonged survival to >200 days (P<0.01). The survival of vvDD-IL15-Rα combined with anti-PD-1 was much more than vvDD-IL15-Rα and vvDD+anti-PD-1 group (108).

In tumor models unresponsive to anti-PD-1 or anti-CTLA4, hIL-17/mIL-12-VV combined with anti-PD-1 or anti-CTLA4 induced a markedly higher CR than viral monotherapy. These data suggest that intratumoral injection of hIL-17/mIL-12-VV has anti-tumor activity in both directly injected and distant tumors and sensitizes tumors to ICIs (150).

In a mouse model of colorectal cancer, AZD4820 combined with anti-PD-L1 increased tumor-specific CD8^+^T cells and tumor PD-L1 expression compared with the AZD4820 alone group; however, it failed to control tumor volume significantly. It is speculated that AZD4820, which carries IL-12, already has an excellent anti-tumor effect. However, the effect of enhancing the expression of CD8^+^T cells and tumor PD-L1 has guiding significance for tumor models that do not respond to ICIs (99).

ASP9801, TBio-6517 and TG6050 are three major OVVs expressing interleukins in clinical trials.

ASP9801 is a vaccinia virus that co-expresses IL-7 and IL-12, and the goal of Phase 1 clinical evaluation is to evaluate its safety and tolerability to determine the dosage for Phase 2 clinical trials. The antitumor activity, objective response rate, pharmacokinetics and viral clearance of ASP9801 as a single agent will be evaluated. And in combination with PD-1 monoclonal antibody pembrolizumab, which has not yet published phase 1 results but has completed enrollment in this trial (NCT03954067).

The Phase 1 trial of TBio-6517, a vaccinia virus that expresses IL-12, aims to determine phase 2 doses and evaluate the efficacy of TBio-6517 in patients with solid tumors through intratumoral or intravenous administration and in combination with pembrolizumab. Similarly, the trial (NCT04301011) completed recruitment but did not publish results.

TG6050, a vaccinia virus that expresses IL-12 and a small amount of Anti-CTLA4, is currently being recruited in a Phase 1 clinical trial (NCT05788926) to explore its dose and toxicity in advanced non-small cell lung cancer.

With the rapid development of ICIs, the great prospect of its combination with OVs is more and more concerned, and many preclinical trials have also proved that the effect of combination therapy is revolutionary. Combining interleukin-armed OVVs with ICIs may be the direction of future clinical trials.

### 4.3 Progress of the combination of interleukin-armed oncolytic vaccinia virus and natural compounds in tumor treatment

Luteolin is known to inhibit tumor growth. Wang et al. applied luteolin to enhance the inhibitory effect of VV-IL-24 on liver cancer. VV-IL-24 combined with luteolin inhibited tumor cells more than either VV-IL-24 or luteolin alone and did not affect normal cells. The tumor volume of mice treated with luteolin and VV-IL-24 was only 105 mm^3^, which was markedly smaller than that recorded in the VV-IL-24 group (1,088 mm^3^), luteolin group (3,080 mm^3^), and PBS group (3,053mm^3^). Hematoxylin and eosin staining confirmed that the tumor tissue in the combined treatment group were damaged more while the liver, kidney and spleen were not. These findings confirmed the better efficacy and safety of the combined treatment (160) (**Table 3**).

**Table 3:** Oncolytic vaccinia virus armed with interleukins combined with other therapies in tumour treatment.

Interleukins	Armed OVV	Other therapy	Cancers	Results	References
IL-6	GLV-1h90	Mitomycin C	Prostatic cancer	thrombocytopenia was delayed Recovery process was accelerated Better anti-tumor effects	(155)
IL-2	VVDD-IL-2-RG	Anti-PD-1 Anti-CTLA4	Colon cancer	metastatic tumors were inhibited	(62)
IL-21	VVL-21	Anti-PD-1	Pancreatic cancer	Better anti-tumor effects	(127)
IL-15	vvDD-IL15-Rα	Anti-PD-1	Colon cancer	Complete cure Survival period was extremely prolonged	(108)
IL-7, IL-12	hIL-17/mIL-12-VV	Anti-PD-1 Anti-CTLA4	Melanoma Colon cancer Lung cancer	Better complete tumor regression	(150)
IL-7, IL-12	ASP9801	Anti-PD-1	Advanced Metastatic Solid Tumors	Recruitment completed	NCT03954067
IL-12	TBio-6517	Anti-PD-1	Advanced Metastatic Solid Tumors	Recruitment completed	NCT04301011
	TG6050	Anti-CTLA4	Non-small-cell Carcinoma	Recruiting	NCT05788926
IL-12	AZD4820	Anti-PD-L1	Colorectal cancer	CD8^+^T cells and PD-L1 expression of tumor cells were increased	(99)
IL-24	VV-IL-24	Luteolin	Liver cancer	Better anti-tumor effects Better safety	(160)

IL, Interleukins, PD-1, Programed death-1, PD-L1, Programed death-Ligand 1, CTLA4, cytotoxic T lymphocyte-associated antigen-4.

## 5 Future perspective

Since the efficacy of OVs as monotherapy is limited; combination therapy is a key strategy to enhance therapeutic outcomes and represents a future trend. At present, we can use OVV as a suitable vector to deliver interleukins to tumors precisely through membrane binding, gene insertion, and other methods. This approach effectively preserves the influence of interleukins on the TME and limits the extremely strong systemic toxicities of many interleukins (161). Interleukins can promote the infiltration of tumor-infiltrating lymphocytes in tumors and upregulate the MHC expression in tumor cells for better surveillance by the immune system (125, 140, 141), thereby enhancing the effect of OVV on immunity. Interleukin-induced memory T cell properties also suppressed tumor recurrence (126, 127). Some interleukins can inhibit macrophages. Without affecting anti-tumor CD8^+^T cells, they reduce the expression of MHC on macrophages and inhibit antiviral CD8^+^T cells. This solves the problem of rapid clearance of OVV by the immunity (82, 83). Furthermore, interleukins can activate immunity to treat distant tumors (125),significantly improving the efficacy of intratumoral injection of OVs in advanced cancer (162–164).

In addition, numerous interleukins have been shown to enhance PD-1/PD-L1 blockade (162–164). Combination of OVVs with PD-1/PD-L1 blockade has also been reported (165, 166). It has been shown that the expression of IL-7 and IL-12 by vaccinia virus can improve the systemic sensitivity to PD-1/PD-L1 blockade (150). Experiments have shown that combining interleukin-armed OVV and PD-1/PD-L1 blockade is more effective and prolongs survival compared with any monotherapy (108, 125). We think that the combination of OVVs expressing interleukins with PD-1/PD-L1 blockade is a promising therapeutic direction.

Moreover, OVV expressing interleukins in combination with chemotherapy is also promising, GLV-1h90 counteracts mitomycin C platelet inhibition by IL-6 to exert a synergistic anti-tumor effect (155). Yu et al. found that cisplatin or gemcitabine potentiated the antitumor effect of GLV-1h68. OVV showed a favorable objective response rate and progression-free survival when combined with platinum-based chemotherapy in patients with platinum-resistant or platinum-refractory ovarian cancer, as shown by the results of a phase II nonrandomized clinical trial (NCT05281471) (167, 168). OVV can further sensitize tumor cells to chemotherapy by promoting the secretion of IFN-I and high-mobility group protein B1 (169).There are few related studies on OVV expressing interleukins combined with chemotherapy, which has huge research prospects.

Another commonly used means of treating tumors is radiotherapy, and studies have shown that radiation combined with OVV has shown considerable antitumor efficacy in several tumor models, such as glioblastoma and pancreatic cancer (170, 171). For the synergistic mechanism, a study by Chen et al. showed that radiotherapy in combination with OVVs can trigger tumor cell necrosis and alter macrophages by releasing damage-associated molecular pattern, generating robust antitumor immunity and enhancing antitumor efficacy (172). And all the above studies demonstrate the potential of radiotherapy and OVVs in combination. We believe that OVVs armed by interleukins combine with radiotherapy deserves further investigation.

Combinations of metabolic modulators with OVs are also promising. It can promote OVs replication and tumor killing by reprogramming tumor cell metabolism, such as enhancing glycolysis inhibition and regulating amino acid and nucleotide metabolism. Simultaneous regulation of immune cell metabolism (such as optimizing T cell glucose and lipid metabolism, NK cell antioxidant pathway) can enhance anti-tumor immune response. Metabolic remodeling combined with oncolytic virotherapy can synergistically enhance the efficiency of viral replication and immune activation, overcome the tumor immunosuppressive microenvironment, and provide a new strategy for cancer immunotherapy. However, it is necessary to balance the relationship between metabolic intervention and antiviral immunity to achieve clinical transformation (173).

A serious problem faced by OVVs is the limited delivery mode just like other OVs. Currently approved OVs are basically given intratumorally, which is undoubtedly not good for patients with advanced systemic metastasis and patients without solid tumors. Intravenous administration of OVs will predictably be necessary. Adenovirus and herpes viruses are widely found in nature, and neutralizing antibodies of both are often present in the human body, so the effect of intravenous administration is greatly reduced. In contrast, OVVs tend to produce neutralizing antibodies after a single intravenous injection which means the development of neutralizing antibodies has limited repeated intravenous administration of OVVs.

To address this problem, inhibitors can be used to block neutralizing antibody production. For example, COX-2 inhibitor can enhance OVVs retention and increase the possibility of repeated dosing by inhibiting neutralizing antibody (174). After prior administration of complement inhibitor,JX-594 (an OVV inserted into GM-CSF),showed an average 10-fold increase in blood infection titers (175).

In addition to the suppression of antiviral immune responses, the implementation of physical shielding strategies through viral encapsulation emerges as a complementary methodology to circumvent rapid immunological clearance and enhance the pharmacokinetic profile of intravenously administered OVVs. Hill et al. employed polyethylene glycol-cholesterol polymer coating to reduce neutralizing antibody binding. Murine studies showed that the polymer coating significantly prolonged the viral circulation time (5-fold increase in plasma drug concentration at 5 minutes), but the improvement in tumor accumulation was limited. This study provides a new strategy for optimizing intravenous delivery of OVV, but it needs to be combined with physical delivery methods to enhance tumor penetration (176). Besides, Nguyen et al. developed a platform based on allogeneic adipose-derived mesenchymal stem cells (AD-MSC). By loading OVVs into AD-MSC successfully protected the virus from inactivation by complement and neutralizing antibodies, and significantly improved virus release efficiency and anti-tumor activity *in vitro* and in animal models. The experiments showed that AD-MSC was more effective than naked virus in inhibiting tumor growth in both immunodeficient and immunocompetent mouse tumor models. In addition, AD-MSC can be used as a genetic engineering vector to insert therapeutic genes, such as fluorescent protein, into noncoding regions without affecting its ability to replicate. This platform provides a new strategy for overcoming the immune barrier of systemic delivery of OVs and has the potential for clinical translation (177).

## 6 Discussion

According to the available evidence, key areas of this field require improvement. Firstly, the preparation process and drug delivery system of the virus should be further optimized to improve its efficacy and reduce side effects. Secondly, a deeper understanding of the mechanisms of virus-host cell interactions is needed to more efficiently control their targeting and safety. In addition, challenges remain regarding the application of OVV armed with interleukins to clinical practice. Firstly, additional long-term safety and efficacy data are needed to support its widespread clinical use. Secondly, the high research and production costs are also important factors hindering its widespread application. A multi-faceted collaboration between policymakers, researchers, pharmaceutical companies, and others is necessary to overcome these challenges.

Based on several studies, we think that the combination of interleukin-modified OVVs with other tumor treatment modalities may attract considerable attention in the future. Interleukins have a very wide range of functions, such as recruiting various immune cells in tumors, enhancing PD-1 inhibitors, countering the side effects of chemotherapy, and activating the systemic immunity. When interleukin is limited to the TME by OVV, introducing another treatment (e.g., ICIs, chemotherapies, and other drugs) can be complementary in maximizing tumor inhibition and preventing tumor recurrence. Most currently ongoing clinical trials investigating armed viruses focus on this direction.

Because of the effects of interleukins and OVVs on the immune system, it was impossible to avoid triggering immune surveillance which and leads to clearance of the virus by immunity. This is the main limitation of the current application of this combination. Some delivery methods have been developed to reduce the clearance of armed viruses. The latest solutions are mainly through polymer encapsulation and the use of MSC as a new carrier to solve the problem of OVV system delivery, In addition, although OVVs armed by interleukins combined with a variety of anti-tumor treatments have good effect in different tumors, it needs huge time and economic cost to use traditional methods to screen one by one. We are concerned about the current use of Artificial Intelligence(AI). We believe that the application of AI technology to predict which interleukins is suitable for OVV modification will not only greatly expand the research ideas, but also improve the research efficiency and reduce the research cost, which is worthy of attention in the future.
